# In Silico Evaluation of HIV Protease and RNA Polymerase Inhibitors as Potential COVID-19 Therapeutics

**DOI:** 10.7759/cureus.69576

**Published:** 2024-09-17

**Authors:** Mahalakshmi Devaraji, Lokeshvar Ravikumar

**Affiliations:** 1 Department of Pharmaceutical Chemistry, Saveetha College of Pharmacy, Saveetha Institute of Medical and Technical Sciences, Chennai, IND; 2 Department of Pharmacology, Saveetha College of Pharmacy, Saveetha Institute of Medical and Technical Sciences, Chennai, IND

**Keywords:** antiviral drugs, covid-19, dock score, molecular docking, sars-cov-2

## Abstract

Background: The COVID-19 coronavirus, also known as the acute respiratory syndrome coronavirus, emerged as a significant global health concern. First identified in Wuhan, China, in December 2019, the virus rapidly spread to over 187 countries due to its high transmissibility. Until an effective treatment or vaccine is developed, preventive measures remain the only mandatory strategy to curb person-to-person transmission.

Aims and objectives: The study aimed to explore potential therapeutic options for COVID-19 by repurposing existing drugs. Specifically, the objective was to evaluate a library of clinically approved or investigational antiviral compounds through docking studies to identify candidates with high binding affinity to COVID-19 proteins.

Materials and methods: The study employed molecular docking techniques using the Maestro interface (Schrodinger Suite, LLC, NY) to assess the interaction of selected compounds with various COVID-19 protein targets. A total of 15 compounds were analyzed for their binding potential to multiple forms of the virus's proteins.

Results: The docking studies revealed that several compounds, particularly HIV protease inhibitors and RNA-dependent RNA polymerase inhibitors, demonstrated strong binding affinities to key COVID-19 enzymes. These interactions suggest their potential as therapeutic candidates for COVID-19 treatment.

Conclusion: The findings from this drug repurposing study highlight the potential of certain existing antiviral agents in the treatment of COVID-19. The identified compounds could serve as promising candidates for further investigation in the ongoing battle against the coronavirus pandemic.

## Introduction

As of 2024, while the global situation regarding COVID-19 has improved significantly due to widespread vaccination efforts and public health measures, certain risks still persist. The emergence of new variants, such as the Omicron subvariants (e.g., XBB.1.5), continues to pose challenges. According to a recent study, these variants have shown the potential for increased transmissibility, although vaccines and boosters remain effective at preventing severe disease, hospitalization, and death. Furthermore, the risk remains higher for specific vulnerable populations, including the elderly, immunocompromised individuals, and those with underlying conditions, such as diabetes mellitus and cardiovascular disease. For instance, a recent CDC report (CDC, 2023) highlighted that despite vaccine coverage, individuals in these categories account for the majority of COVID-19-related hospitalizations and deaths, especially in regions with low booster uptake. However, for the general public, the risk of severe outcomes has reduced significantly due to the high effectiveness of vaccines, improved treatment protocols, and public health interventions. Continued vigilance, booster campaigns, and adaptive strategies are essential to mitigate the evolving risks posed by newer variants [1,2]. This respiratory infection results in fever, exhaustion, a dry cough, myalgia, and breathlessness.COVID-19 can result in severe complications, including pneumonia and organ failure, potentially leading to death. Pneumonia can arise as a result of this. Acute respiratory distress syndrome (ARDS) can be a complication. A severe lung inflammation is characterized as respiratory distress syndrome [3].

Coronavirus has an incubation period of one to 14 days, during which the virus replicates in the body before symptoms appear. The incubation period is not related to the virus’s ability to "breathe" or oxygen levels, as viruses do not require oxygen to survive or replicate [4]. The severity of symptoms differs from one patient to the next. Symptom severity varies from one patient to the next. Because of their low or compromised immune systems, The elderly and pediatric populations are at heightened risk of severe COVID-19 outcomes, and patients with comorbidities such as bronchial asthma and diabetes mellitus are at higher risk of severe illness. The worst outbreak occurred in China particularly in Wuhan [5-6]. Due to its rapid spread and an estimated basic reproduction number (R0) of approximately 2.2, severe acute respiratory syndrome coronavirus 2 (SARS-CoV-2) has shown significant potential for transmission, the WHO labeled this occurrence a public health emergency of international concern in January 2020. With more than 266,071 confirmed cases and more than 11,000 confirmed deaths as of March 20, 2020, it had spread to around 187 countries, resulting in a case fatality rate (CFT) of 4.4 [7]. The causative agent of COVID-19 is SARS-CoV-2, a novel SARS-CoV-2. The Middle-Eastern respiratory syndrome coronavirus (MERS-CoV) and SARS-CoV-2 are related viruses [8-9]. SARS-CoV-2 primarily affects the lower respiratory system, but it can also impact other organs and systems in the body.

SARS-CoV-2 primarily binds to the ACE2 receptor, which is highly expressed in the lungs, gastrointestinal system, liver, heart, and other organs. After binding, the virus penetrates the cytoplasmic lung epithelial cells, releases its nucleocapsid, and hijacks the host's cellular machinery to replicate. The Coronaviridae family includes the single-stranded, positive-strand RNA viruses known as SARS-CoV-2. SARS-CoV and SARS-CoV-2 share a lot of structural similarities. There are 14 interaction residues in the SARS family, with eight of them being specific to SARS-CoV-2. Of note, this family's binding residues interact with angiotensin-converting enzyme-2 (ACE-2) directly [10]. Various preventive methods have been advocated by healthcare authorities all around the world. Quarantining sick patients, conducting thorough testing and detecting probable victims quickly, wearing adequate masks, and washing hands frequently will all help to prevent and control this terrible disease [11].

While vaccines and several treatment options are now available for COVID-19, SARS-CoV-2 initially posed a significant threat due to its high transmission rate. A symptomatic person could infect more than two others. Currently, scientists continue to explore new therapeutic uses for existing medications alongside vaccine development to improve treatment outcomes. Experts in the field have identified several broad-spectrum antiviral drugs as potential therapeutic options for COVID-19, including nucleoside analogs and HIV enzyme inhibitors. Key therapeutic targets in COVID-19 include RNA-dependent RNA polymerase (RdRp) and the ACE2 receptor. Antiviral medications such as remdesivir, oseltamivir, ganciclovir, favipiravir, lopinavir, and ritonavir have been evaluated in clinical trials. Remdesivir has been granted full FDA approval, and newer therapies like Paxlovid (nirmatrelvir and ritonavir) and Molnupiravir have also been introduced for treating COVID-19, especially in high-risk patients.

Early studies suggested that hydroxychloroquine might be effective in treating COVID-19 [12], but large-scale trials, such as the Recovery trial, have since demonstrated that hydroxychloroquine is not effective. Current treatment strategies have shifted toward newer antiviral drugs, such as Paxlovid (nirmatrelvir and ritonavir) and Molnupiravir, which have shown promise in reducing the severity of symptoms in high-risk patients. These newer treatments should be discussed further in the context of their clinical efficacy and usage. To find an appropriate small chemical to treat the life-threatening coronavirus disease, docking experiments were undertaken on the binding pocket of COVID-19.

## Materials and methods

Molecular modeling platform

The Schrodinger software (Maestro 11.4, Schrodinger 2021-2, Schrodinger LLC, New York, USA) was used for the computational molecular docking study.

Preparation of ligands

A total number of 15 compounds were chosen for the molecular docking study experiments and find out the strong binding capacity for COVID-19, the medications starting with the introduction of antiviral therapies in 1960 through the most recent trends in therapeutics being tested in clinical trials [13-14]. The 3D chemical structure of the compounds was retrieved from the PubChem database [15]. By introducing hydrogen (polar group), removing salt and hetero atoms, and ionizing at pH, ligand preparation using the tool Lig Prep (Schrodinger LLC, New York, USA, 2009) was utilized to synthesize ligands and generate three-dimensional structures of the ligand. From the Maestro design panel, ligand optimizations in 3D and geometry were done (7.2) [16-17].

Grid generation and protein structure preparation

The Protein Data Bank (PDB) identifiers for the structures discussed are 6B7N, 7BAJ, 6VWW, and 7CAB resolutions between 1 and 3 were chosen and successfully downloaded from Protein Data Bank (http://www.rscb.org) to address the current status of COVID-19 protein structure [18-21]. In the Maestro panel, the protein preparation wizard is used to prepare protein structures. The protein building blocks and hydrogen atoms were included in this protein synthesis. The water molecules are eliminated from the het group by three units [22-23]. Finally, the protein structure is minimized by the 0PLS-2005 force field in the Maestro panel [24]. Using the "Glide's Receptor Grid Generation" module with a computational cubic box of 10Å×10Å×10Å, an additional receptor grid box was generated at attribute active sites (with a radius of 30Å) of co-crystallized ligands [25].

Molecular docking studies 

Molecular docking, a method for structure-based drug design, exposes the critical interconnections between a targeting protein's amino acid residues and its low-energy ligands [26]. The scoring system utilized to determine the binding effectiveness with the receptor serves to identify the minimum interactions of the ligands. Gliding over any limitations will spread them. It was able to forecast the binding interactions and ligands' effectiveness as COVID-19 target inhibitors with the aid of flexibility binding and the Glide Standards precision (SP) approach [27]. The energy evaluation was carried out using the dock score. We used the Maestro interface to analyze docked ligands [28].

Dock score= a× vdW+ b×Coul+Hbond+ Metal + Lipo+ BuryP+RotB +Site,

where a and b are the co-efficient constants for vdW and Coul, respectively; vdW = van der Waals energy; Coul = Coulomb energy; Hbond = hydrogen bonding with receptor; Metal = binding with metal; Lipo = constant term for lipophilic; BuryP = buried polar group penalty; RotB = rotatable bond penalty; and Site = active site polar interaction [29].

## Results

Docking studies

Molecular docking analyses on the binding of the COVID-19 enzyme, the specific enzyme being referenced is the RNA-dependent RNA polymerase (RdRp) to 15 drugs were carried out to uncover a promising option for treating COVID-19 (PDB ID: 6B7N, 7BAJ, 7CAB, 6VWW). Among the antiviral drugs discussed, some are already available on the market, while others are still undergoing clinical trials. For example, remdesivir, an antiviral drug currently approved for emergency use, is used to treat severe COVID-19 infections. Oseltamivir, another antiviral, is used for treating influenza. By contrast, drugs like Molnupiravir and Paxlovid (nirmatrelvir and ritonavir) are newer treatments that have recently received emergency use authorization for COVID-19 but are still subject to ongoing evaluation for their full approval and long-term efficacy. These drugs target various viruses including smallpox, influenza, hepatitis, HIV, herpes, cytomegalovirus, and Ebola. These medications work in a variety of ways, including inhibiting enzymes such as reverse transcriptase, proteases, and DNA polymerase. Table 1 shows the medications that were examined for docking studies.

**Table 1:** Antiviral drugs and their target enzymes for COVID-19

No	Drugs
1	Acarbose
2	Camostat
3	Ciprofloxacin
4	Favipiravir
5	Hydroxychloroquine
6	Ivermectin
7	Lopinavir
8	Migilitol
9	Nafamostat
10	Nitazoxanide
11	Oseltamivir
12	Remdeivir
13	Ritonavir
14	Serotonin-Norepinephrine Reuptake Inhibitor
15	Umifenovir

The coronavirus target protein was docked with each of the 15 drugs, and the results were ranked by the dock score. It is hypothesized that compounds with a docking score of 6.5 or lower may exhibit strong inhibitory potential against COVID-19 as shown in Table 2. This cutoff is based on empirical data and literature where lower docking scores typically correspond to higher binding affinities and potentially greater effectiveness in inhibiting the target protein. However, the selection of this cutoff should be interpreted in the context of additional validation through experimental assays and comparative studies, as docking scores alone may not fully predict biological activity, it also can be used to perform a comparison analysis. The list of active compounds acquired from docking experiments is represented in Table 1. These compounds have a docking score of less than 7. A total of 15 compounds were found to bind to COVID-19 structures with PDB IDs 6B7N, 7BAJ, 7CAB, and 6VWW.

**Table 2:** Results of a comparative docking investigation on COVID-19 enzymes

Serial no.	Drug	Dock score			
		6B7N	7BAJ	7CAB	6VWW
1	Acarbose	-5.589	-	-5.562	-2.720
2	Camostat	-2.269	-2.440	-2.967	-6.083
3	Ciprofloxacin	-4.146	-	-4.540	-5.618
4	Favipiravir	-4.034	-4.859	-3.832	-4.024
5	Hydroxychloroquine	­-4.113	-1.010	-5.026	-5.035
6	Ivermectin	-3.505	-	-2.478	-7.190
7	Lopinavir	-4.142	-	-3.913	-3.334
8	Migilitol	-5.114	-	-5.080	-5.525
9	Nafamostat	-2.518	-	-4.547	-6.207
10	Nitazoxanide	-3.275	-3.978	-4.250	-4.448
11	Oseltamivir	-3.627	-	-3.330	-3.199
12	Remdeivir	-4.591	-	-4.737	-4.448
13	Ritonavir	-4.494	-	-5.413	-6.049
14	Serotonin	-5.729	-4.788	-5.899	-5.672
15	Umifenovir	-3.235	-1.022	-3.275	-4.040

Camostat, favipiravir, hydroxychloroquine, nitrozoxanide, serotonin, and umifenovir were discovered to interact with >2 protein structures of coronavirus out of 15 compounds tested. Favipiravir, an anti-HIV medication, is capable of attaching to any viral protein complexes with a docking score of under -6.5. In-silico docking studies revealed the best results for all HIV protease inhibitors. Apart from protease inhibitors, COVID-19 enzyme inhibitors include camostat, hydroxychloroquine, nitrozoxanide, and serotonin.

**Figure 1:**
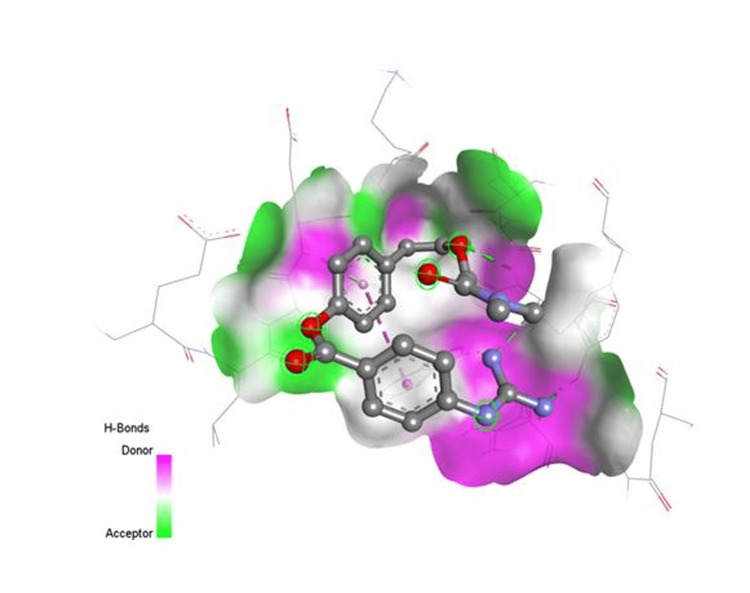
Docking interaction of camostat with 6VWW

**Figure 2:**
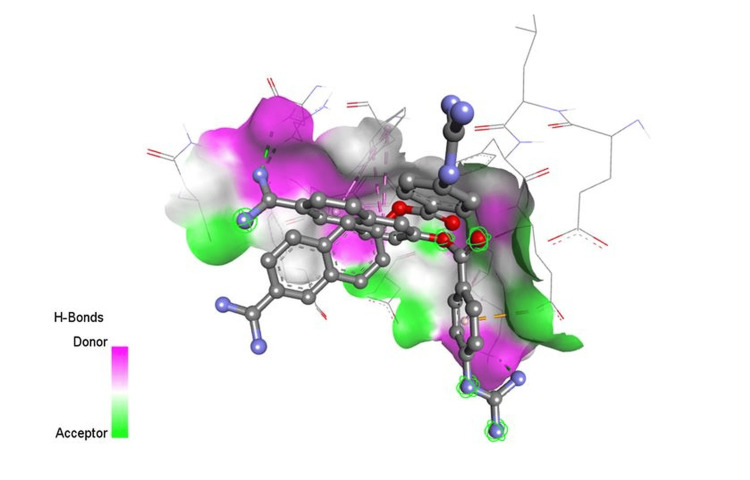
Docking interaction of nafamostat with 6VWW

**Figure 3:**
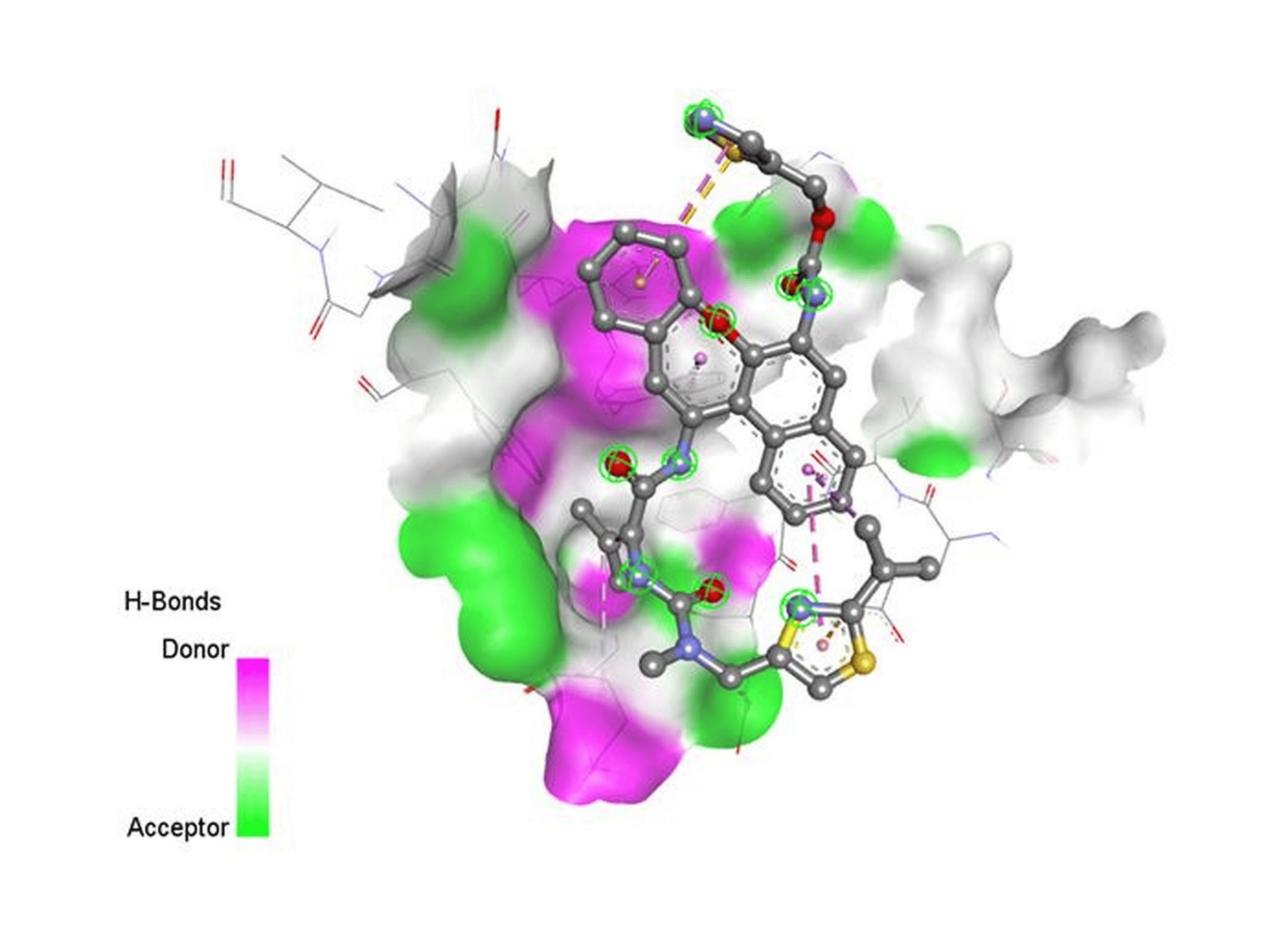
Docking interaction of ritonavir with 6VWW

In the instance of remdesivir, interactions with COVID-19 are similar to favipiravir, but remdesivir has one significant hydrophilic interaction. Because hydroxychloroquine had a high bind score with the COVID-19 enzyme, it was chosen. These interactions may increase the clinical potency of the SARS-COV-2 therapeutic agents. The medications are useful for corona infections based on these docking data. Figures 1-3 show an interaction between camostat, nafamostat, and ritonavir strongly bind to the 6VWW protein.

## Discussion

The current study explored the potential of 15 drugs, including both market-approved medications and those under clinical investigation, as inhibitors of key COVID-19 enzymes. Through molecular docking analyses, we identified several compounds with promising binding affinities to COVID-19 protein structures, specifically PDB IDs 6B7N, 7BAJ, 7CAB, and 6VWW. The docking scores provided a quantitative assessment of the binding strength, with a score of -6.5 or lower indicating potent inhibitory potential against the SARS-CoV-2 enzyme.

Notably, our results highlighted several drugs with substantial binding affinities, including camostat, favipiravir, hydroxychloroquine, nitazoxanide, serotonin, and umifenovir. These compounds were found to interact with multiple COVID-19 protein structures, suggesting broad-spectrum activity against the virus. Favipiravir, an anti-HIV drug, demonstrated particularly strong binding across various viral protein complexes, with docking scores consistently below -6.5. This is in line with the emerging role of HIV protease inhibitors in COVID-19 treatment, where they may disrupt the virus's ability to replicate by inhibiting essential proteolytic processes.

Paxlovid (nirmatrelvir and ritonavir) and Molnupiravir are two notable antiviral treatments that have recently been granted emergency use authorization for COVID-19. Paxlovid is a combination of nirmatrelvir, a protease inhibitor, and ritonavir, which helps increase the levels of nirmatrelvir in the body. It is used for treating mild-to-moderate COVID-19 in high-risk patients to prevent progression to severe disease. Molnupiravir is a nucleoside analog that inhibits viral replication by introducing errors into the viral RNA. Both drugs have shown promise in clinical trials and have been included in the discussion section of the updated manuscript, detailing their mechanisms of action, clinical trial results, and current indications [30].

Interestingly, remdesivir, another widely studied antiviral agent, exhibited binding characteristics similar to favipiravir but included a significant hydrophilic interaction that could influence its clinical effectiveness. Hydroxychloroquine, despite controversy in clinical settings, showed high binding scores, reinforcing its potential as a COVID-19 enzyme inhibitor. This supports the hypothesis that hydroxychloroquine's mechanism of action may extend beyond immunomodulation, involving direct interactions with viral enzymes [31-33].

The docking studies also provided insights into the binding behavior of other compounds, such as camostat, which, along with nafamostat and ritonavir, exhibited strong interactions with the 6VWW protein structure. These findings align with the mechanism of action of protease inhibitors, which prevent the virus from processing its polyproteins, thereby inhibiting viral replication [34-36].

The identification of these compounds as potential inhibitors of SARS-CoV-2 enzymes underscores the utility of drug repurposing in the fight against COVID-19. By leveraging existing therapeutics, we can accelerate the development of effective treatments and reduce the time required for clinical translation. Moreover, the results of this study provide a rationale for further in vitro and in vivo studies to validate the antiviral efficacy of these compounds and explore their potential integration into COVID-19 treatment regimens.

Limitations of the study

Despite the promising findings, several limitations should be considered when interpreting the results of this study. First, while molecular docking provides valuable insights into potential drug-protein interactions, it is an in silico approach that may not fully replicate the complex dynamics of biological systems. The binding affinities observed in docking studies do not always translate into actual pharmacological efficacy, as bioavailability, metabolic stability, and off-target effects play critical roles in drug activity. Second, the study focused solely on the interaction of drugs with key COVID-19 enzymes, without considering other viral components or host factors that could influence viral replication and pathogenesis. The multifaceted nature of COVID-19 implies that inhibition of viral enzymes alone may not be sufficient to halt disease progression, and a broader therapeutic approach may be necessary.

In addition, the docking scores, while indicative of binding potential, do not account for potential side effects or toxicity profiles of the drugs when used in the context of COVID-19 treatment. Clinical relevance can only be fully assessed through rigorous in vitro, in vivo, and clinical trials. Finally, the current study did not explore the potential for drug resistance, a critical consideration when repurposing antiviral agents for COVID-19 therapy. Addressing these limitations will require further experimental validation and clinical studies to determine the true therapeutic potential of the identified compounds. Nonetheless, the findings provide a valuable starting point for future research to repurpose drugs for COVID-19 treatment.

## Conclusions

In summary, while lopinavir, ritonavir, and favipiravir have shown potential in antiviral therapy, their effectiveness against COVID-19 is still subject to ongoing research, and broader conclusions about their utility must await further clinical evidence. Acarbose and camostat, two other compounds that have been discovered as COVID-19 inhibitors, interact with the 7CAB, 6VWW, and 6B7N proteins and have docking scores of -5.569, -2.720, and -5.589, respectively. According to this study, glucosidase inhibitors show potential activity against COVID-19 enzymes. The docking scores of nitrozoxanide with proteins 6B75, 7BAJ, 7CAB, and 6VWW are -3.275, -3.978, -4.250, and -4.448, respectively. The nitrozoxanide drug treatment available for antiprotozoal by interfering with pyruvate ferredoxin/flavodoxin oxidoreductase reaction is a promising possibility for the treatment of coronavirus disease. According to our research, COVID-19 enzymes are also inhibited by other medications such as acarbose, camostat, and serotonin in addition to protease inhibitors. Future research and clinical trials aimed at studying the effectiveness of these antiviral medications may lead to improved strategies for managing COVID-19 symptoms and enhancing patient outcomes. Continued investigation into these and other potential treatments will be crucial for developing more effective therapies and optimizing COVID-19 management.
